# Anti-inflammatory effects of millet bran derived-bound polyphenols in LPS-induced HT-29 cell via ROS/miR-149/Akt/NF-κB signaling pathway

**DOI:** 10.18632/oncotarget.20216

**Published:** 2017-08-12

**Authors:** Jiangying Shi, Shuhua Shan, Hanqing Li, Guisheng Song, Zhuoyu Li

**Affiliations:** ^1^ Institute of Biotechnology, Key Laboratory of Chemical Biology and Molecular Engineering of National Ministry of Education, Shanxi University, Taiyuan 030006, PR China; ^2^ College of Life Science, Shanxi University, Taiyuan 030006, China; ^3^ Department of Medicine, Division of Gastroenterology, University of Minnesota Medical School, Minneapolis, Minnesota, MN 55455, USA

**Keywords:** foxtail millet bran, bound polyphenols, ROS, miR-149, anti-inflammation

## Abstract

The pro-inflammatory and anti-inflammatory maladjustment has been acknowledged as one of the chief causations of inflammatory diseases and even cancers. Previous studies showed that plant-derived polyphenolic compounds were the most potent anti-oxidant and anti-inflammatory agents among all natural compounds. The present study indicates that bound polyphenols of inner shell (BPIS) from foxtail millet bran can display anti-inflammatory effects in LPS-induced HT-29 cells and in nude mice. Mechanistically, BPIS restrained the level of various pro-inflammatory cytokines (IL-1β, IL-6, IL-8), and enhanced the expression level of anti-inflammatory cytokine (IL-10) by blocking the nuclear factor-kappaB (NF-κB)-p65 nuclear translocation. Further, we found the elevated miR-149 expression by BPIS-induced ROS accumulation, directly targeted the Akt expression to block NF-κB nuclear translocation. Taken together, these novel findings provide new insights into the development of BPIS as an anti-inflammatory agent via the signaling cascade of ROS/miR-149/Akt/NF-κB axis.

## INTRODUCTION

Clinically, the postoperative infections are a pressing public health challenge, and are primarily attributed to bacteria and viruses. Lipopolysaccharides, a major component of gram-negative bacterial cell wall play a crucial role to provoke the immune response [1], which lead to chronic inflammation and cancer recurrence particularly colorectal cancer (CRC) [2, 3]. In general, inflammation is a complex physiological response of body tissues to disease causing irritants, which aims to remove the harmful stimuli and promotes wound healing [4]. However, long-term inflammatory reactions are considered as the new malignant phenotype of cancer cells and diffusely approved that chronic inflammation plays a pivotal role in the early stage of cancer [5-7]. Furthermore, the maladjustment of pro-inflammatory and anti-inflammatory cytokines, including IL-1β, TNF-α, IL-6 and IL-8, are inflammatory biomarkers in animal experiments and clinical studies [8]. Therefore, controlling LPS-induced inflammatory factors might reduce CRC risk and improve the clinical prospect.

Foxtail millet (*Setaria italica*), one of the oldest cultivated cereals in China, contains an abundance of vitamins, minerals and proteins containing significant abundance of essential amino acids profile [9, 10]. Foxtail millet bran, the by-product of foxtail millet processing, is an excellent source of phytochemicals, among which polyphenols exhibit a wide range of pharmacological and medicinal characteristics [11]. Content of plant-derived polyphenols such as ferulic acid, coumaric acid, cinnamic and gentisic acid of finger millet, pearl millet, teff millet, fonio millet and foxtail millet have been reported [12]. Naturally occuring polyphenols, existing mainly as bound forms, were found to possess anti-oxidant, anti-tumor, anti-inflammatory and immunomodulation effects [13]. Such effects were observed both *in vivo* and *in vitro* and were believed due to their free radical scavenging [14-16]. Whereas, there is a growing evidence indicated that bound polyphenols could also act as pro-oxidant chemical messengers in tumor cells and normal cells [17, 18]. Moreover, it was shown that BPIS possesses a broad-spectrum anti-tumor property and such property was associated with elevation of ROS [9]. However, the mechanism how ROS levels are linked with anti-inflammation property is not known in HT-29 cells.

It has been reported that ROS is able to activate the p53 tumor suppressor protein which regulates downstream gene expression by acting as a transcriptional factor [19]. Activation of p53 results in inhibition of miRNA expression [20, 21]. MiRNAs function as either tumor suppressor gene or oncogene depending on their target genes. The regulation of target gene expression by MiRNA is achieved by direct binding to the mRNA of target gene [22]. Therefore, ROS is able to affect the expression of specific miRNAs through its ability to regulate p53 activity. Although it has been found that BPIS causes increased production of ROS in cancer cells, whether the increase of cellular ROS can affect the expression of a particular miRNA and its downstream target genes is poorly understood.

Our results showed that BPIS could reduce the levels of pro-inflammatory cytokines (IL-1β, IL-8 and IL-6) and promoted the expression of anti-inflammatory cytokine (IL-10) *in vitro* and *in vivo* by blocking NF-κB nuclear translocation. Mechanistically, BPIS treatment of HT-29 cells promoted the ROS accumulation leading to the increase of miR-149 expression. In addition, we found that miR-149 directly targeted the 3′-UTR of Akt to inhibit its downstream NF-κB activation, and then attenuated expression of pro-inflammatory molecules in LPS-induced HT-29 cells. Hence, the present data suggest that the millet bran-derived BPIS is a potential anti-inflammatory therapeutic agent for attenuating LPS-mediated inflammation in CRC.

## RESULTS

### Inhibitory effects of BPIS on the pro-inflammatory cytokines in LPS-induced HT-29 cells

**Figure 1:**
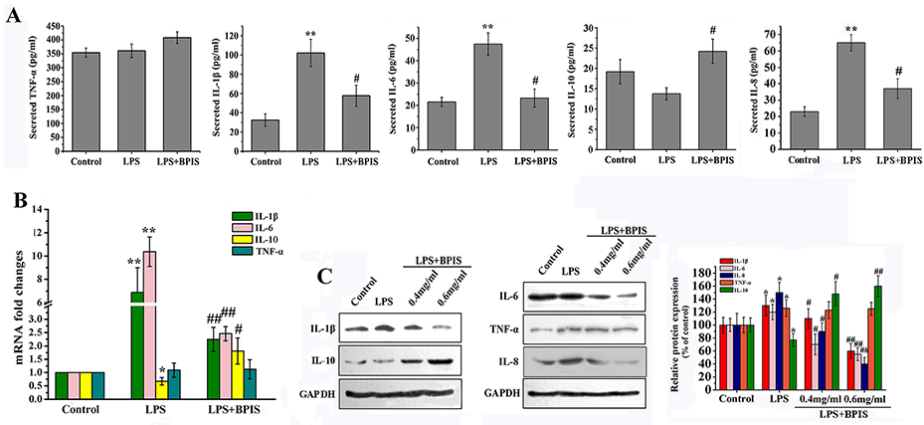
Effects of BPIS on LPS induced inflammation **(A)** Cells were incubated with or without BPIS (0.6 mg/mL) in the presence of LPS (0.01 μg/ml) for 24 h. The levels of secreted TNF-α, IL-1β, IL-6, IL-8 and IL-10 at 24 h in the cell culture medium were analyzed by ELISA. Data were presented as mean ± SEM (n=4, **p<0.01 vs. control, # p<0.05 vs. LPS). **(B)** Total RNAs were prepared from HT-29 cells. Cells were treated with 0.01 μg/ml LPS for 24 h, and then stimulated with or without 0.6 mg/ml BPIS for 24 h. The mRNA expression levels of IL-1β, IL-6, IL-10 and TNF-α were carried out by RT–PCR and expression levels were normalised to GAPDH. Data were presented as mean ± SEM (n=3,* p<0.05 vs. control, **p<0.01 vs.control, # p<0.05 vs. LPS, ## p<0.01 vs. LPS). **(C)** Cells were cultured with different concentrations of BPIS (0, 0.4 mg/ml and 0.6 mg/ml) in the absence or presence of 0.01 μg/ml LPS for 24 h. The protein expression levels of IL-1β, IL-6, IL-8, IL-10 and TNF-α were measured by western blot. Data were presented as mean ± SEM (n=3, *p<0.05 vs. control, # p<0.05 vs. LPS, ## p<0.01 vs. LPS)

LPS initiates inflammatory responses and develop inflammation by expressing pro-inflammatory cytokines, including TNF-α, IL-1β, IL-6 and IL-8 [23]. Therefore, we investigated whether BPIS could suppress pro-inflammatory cytokines induced by LPS in HT-29 cells. The results showed that BPIS and LPS co-treatment significantly inhibited the secretion of pro-inflammatory cytokines, including IL-1β level from 102.51±15.02 pg/ml to 56.44±8.62pg/ml, IL-6 from 48.31±7.15 pg/ml to 23.06±3.58 pg/ml, IL-8 from 65.36±5.03 pg/ml to 37.88±4.72 pg/ml and the increased secretion of IL-10 from 13.91±2.84 pg/ml to 23.47±3.41 pg/ml in LPS-induced HT-29 cells, yet no significant change has found in TNF-α (Figure 1A). Followed by BPIS and LPS cotreatment, the expression level of inflammatory factors was measured at both the mRNA and protein levels via RT-PCR and western blot (Figure 1B and 1C). We found that BPIS significantly (*p*<0.05) decreased the expression of LPS-induced IL-1β, IL-6 and IL-8 (*p*<0.05), while, increase the expression level of IL-10, still TNF-α expression remained unchange. Collectively, this data indicates that BPIS can potentially inhibit the production of pro-inflammatory cytokines in LPS-induced HT-29 cells.

### BPIS attenuates NF-κB activation in LPS-induced HT-29 cells

**Figure 2:**
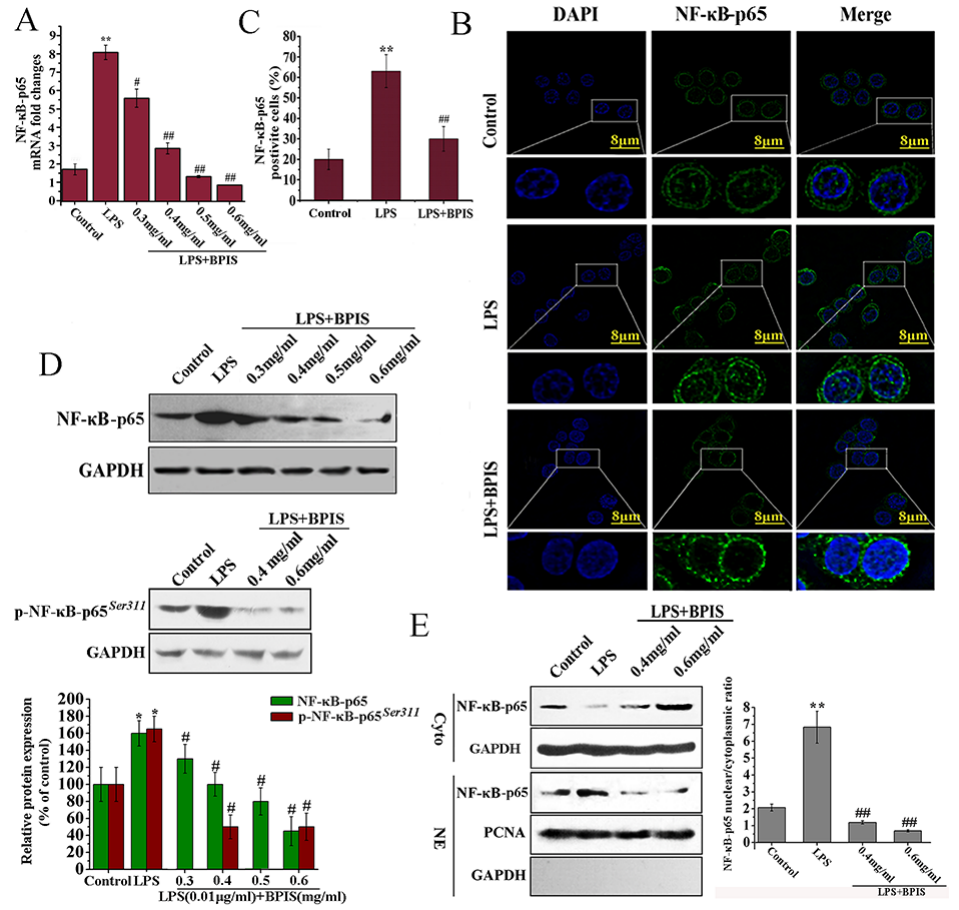
BPIS prevented nuclear translocation of NF-κB in LPS treated HT-29 cells **(A)** Cells were stimulated with LPS (0.01 μg/ml) for 24 h, and then treated with or without various concentrations of BPIS (0.3 mg/ml, 0.4 mg/ml, 0.5 mg/ml and 0.6 mg/ml) for 24 h. MRNA levels of NF-κB-p65 were determined by RT–PCR using internal control (GAPDH). Data were presented as mean ± SEM (n=3, **p<0.01 vs. control, # p<0.05 vs. LPS, ## p<0.01 vs. LPS) **(B)** Cells were incubated with or without BPIS (0.6 mg/ml) in the presence of LPS (0.01 μg/ml) for 24 h. Green staining of NF-κB-p65 was present in the nucleus but absent in the cytoplasm of HT-29 cells treated for 24 h with 0.01 μg/ml LPS alone. Dominant green cytoplasmic localization of NF-κB-p65 was observable in cells treated by adding LPS with 0.6 mg/ml BPIS. **(C)** The percentage of NF-κB-p65 positive cells in ten random fields of the same size. Data were presented as mean ± SEM (n=5, **p<0.01 vs. control, ## p<0.01 vs. LPS). **(D)** Western blot to evaluate the expression level of NF-κB-p65 and p-NF-κB-p65(Ser311) in cell lysates using relevant antibodies. GAPDH was used as a standard control. Data were presented as mean ± SEM (n=3, *p<0.05 vs. control, # p<0.05 vs. LPS). **(E)** The cells untreated and treated LPS alone or treated by adding LPS with 0.6 mg/ml BPIS. Western blot assays of NF-κB-p65 expression in nuclear and cytosolic. PCNA and GAPDH were the loading controls, and the presence of GAPDH in nuclear extracts illustrated whether cytoplasmic protein was contaminated. Data were presented as mean ± SEM (n=3, **p<0.01 vs. control, ## p<0.01 vs. LPS).

NF-κB plays a critical role to regulate the expression of various inflammatory cytokines [24]. Because NF-κB-p65 can contribute directly to DNA binding in the NF-κB complex. We evaluated the expression of NF-κB-p65 followed by BPIS treatment in LPS-induced HT-29 cells. The results showed that BPIS highly (*p*<0.01) suppressed the NF-κB-p65 expression on both the mRNA and protein levels in a dose-dependent manner (Figure 2A and 2D). A substantial number of studies have shown that LPS activates NF-κB by initiating nuclear translocation of NF-κB-p65 [25]. Therefore, we examined the NF-κB-p65 localization after BPIS treatment in LPS-induced HT-29 cells by immunofluorescence (Figure 2B and 2C), BPIS treatment significantly (*p*<0.01) reduced the amount of NF-κB-p65 in the nucleus and increased NF-κB-p65 expression in the cytosol in LPS-induced HT-29 cells. Simultaneously, the expression of p-NF-κB-p65 (Ser311) had been restrained (Figure 2D). Furthermore, the localization of NF-κB-p65 was determined by western blot analysis. Compare to the LPS individual treatment, BPIS and LPS co-treatment drastically decreased NF-κB-p65 nuclear localization, with high dose of BPIS (Figure 2E).

### BPIS inhibits the NF-κB expression through blocking the phosphorylation of Akt

**Figure 3:**
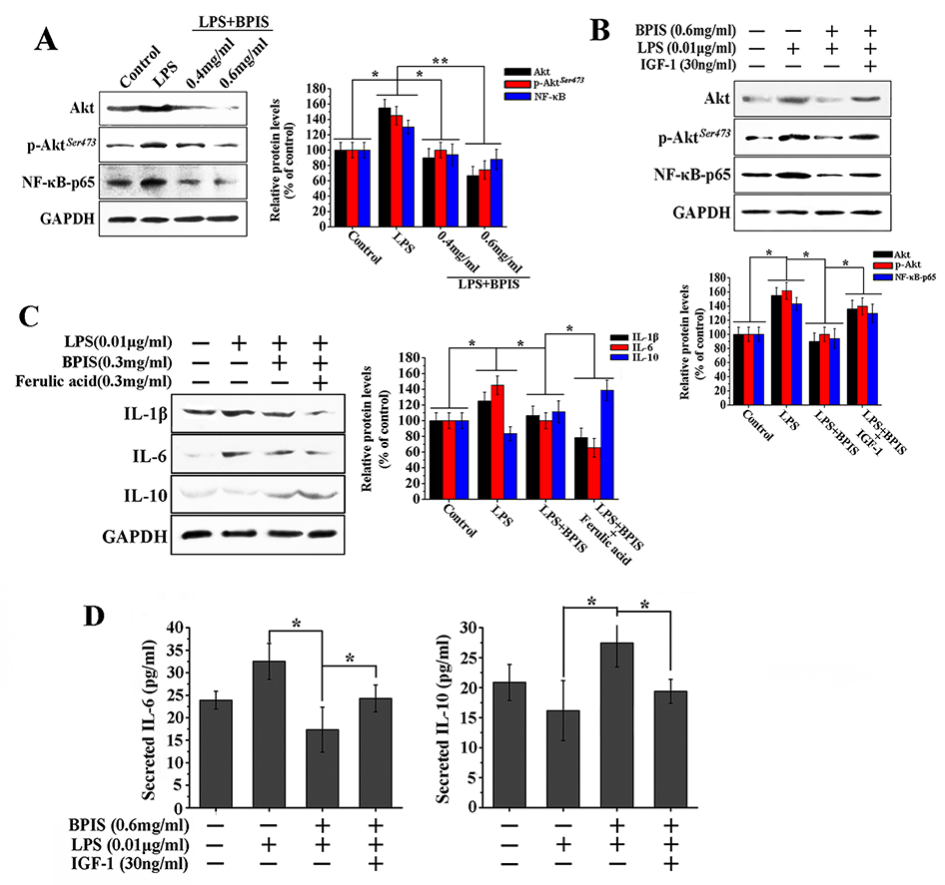
BPIS suppressed LPS-induced NF-κB expression by reduced phosphorylation of Akt in HT-29 cells **(A)** HT-29 cells were treated for 24 h with 0.01 μg/ml LPS alone or together with various concentrations of BPIS (0.4 mg/ml and 0.6 mg/ml). The expression levels of total Akt, p-Akt and NF-κB-p65 were measured by western blot. Data were presented as mean ± SEM (n=3, *p<0.05, **p<0.01). **(B)** The cells untreated and treated 0.01 μg/ml LPS alone or treated by adding LPS with 0.6 mg/ml BPIS and 30 ng/ml IGF-1 (Akt activator). The protein expression levels of total Akt, p-Akt and NF-κB-p65 were measured by western blot. Data were presented as mean ± SEM (n=3, *p<0.05,). **(C)** The cells untreated and treated 0.01 μg/ml LPS alone or treated by adding LPS with 0.3 mg/ml BPIS and 0.3mg/ml ferulic acid. The protein expression levels of IL-1β, IL-6 and IL-10 were measured by western blot. Data were presented as mean ± SEM (n=3, *p<0.05). **(D)** HT-29 cells untreated and treated with 0.01 ng/ml LPS alone or treated by adding LPS with 0.6 mg/ml BPIS and 30 ng/ml IGF-1 (Akt activator). The levels of secreted IL-6 and IL-10 at 24 h in the cell culture medium were analyzed by ELISA. Data were expressed as mean ± SEM (*n* = 4, * p < 0.05).

The grounds that the PI3K/Akt pathway involved in the regulation of NF-κB activity, suggesting a mechanistic link between Akt and NF-κB signaling axis [26]. To determine whether the suppression of NF-κB by BPIS-induced related to Akt signaling pathway, the effects of BPIS on total Akt, Akt phosphorylation and NF-κB-p65 were tested. We found that BPIS and LPS co-treatment significantly declined the total Akt, Akt phosphorylation and NF-κB-p65 as compared to the LPS individual treatment in a dose-dependent manner (Figure 3A). Furthermore, followed by PI3K/Akt activator (IGF-1) and BPIS co-treatment, the total Akt, Akt phosphorylation and NF-κB-p65 protein level were found markedly reversed in LPS-induced HT-29 cells (Figure 3B). Further, we examined the effects of IGF-1 and BPIS co-treatment on the levels of IL-6 and IL-10 secretion. As expected, the levels of BPIS-inhibited IL-6 and IL-10 secretion were significantly reversed in LPS-stimulated HT-29 cells (Figure 3D). In addition, ferulic acid the predominant millet-derived polyphenol found in the bound form [27]. Therefore, we measured the changes of IL-1β, IL-6 and IL-10 after BPIS and ferulic acid co-treatment. BPIS and LPS co-treatment significantly decreased the IL-1β, IL-6 and increased IL-10 compared with the LPS single treatment in a dose-dependent manner (Figure 3C). These results indicate that BPIS displays the anti-inflammatory effects by blocking the expression of Akt and its downstream NF-κB signaling pathway.

### miR-149 directly inhibits Akt expression

**Figure 4:**
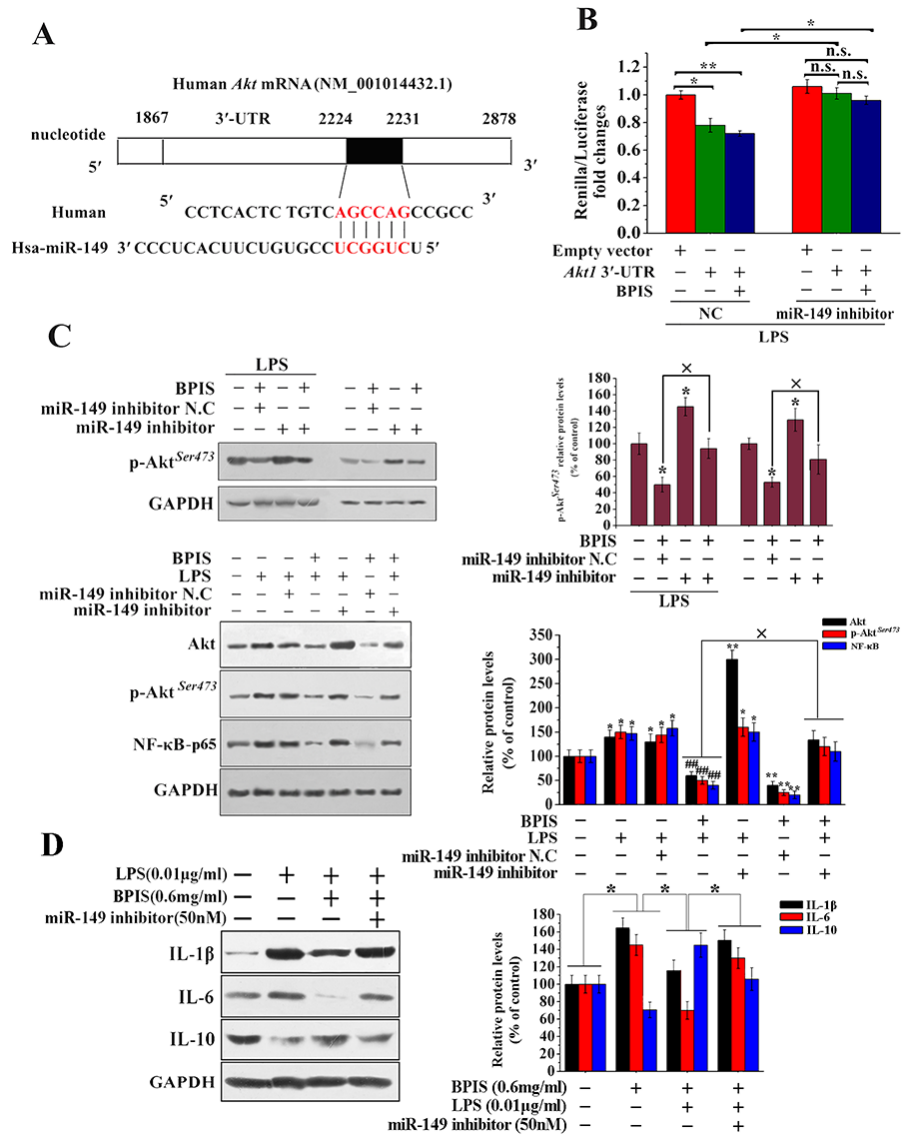
miR-149 directly inhibited Akt expression **(A)** Predicted miR-149 target sequence of the Akt 3’ -UTR used bioinformatics analysis. **(B)** Luciferase reporter assay. We co-transfected 50 ng of psiCHECK-2-Akt1 3’ -UTR plasmid and 20 μM of miRNA-149 inhibitor or miRNA-149 inhibitor N.C into HT-29 cells. The N.C treatment group inhibited the luciferase activity of the Akt1 3’-UTR construct. By contrast, miRNA-149 inhibitor increased miR-N.C-inhibited luciferase activity of the Akt1 3’-UTR construct (* p<0.05, ** p<0.01). Each bar represents the mean ± SEM of three independent experiments. **(C)** Western blot analysis of total Akt, p-Akt and NF-κB-p65 levels in BPIS treated with LPS and miR-149 inhibitors. Data were presented as mean ± SEM (n=3, *p<0.05 vs. control, ## p<0.01 vs. LPS, ×p<0.05). **(D)** The effect of pretreatment with miRNA-149 inhibitor for 24 h on the BPIS-treated the expression of IL-1β, IL-6 and IL-10 in LPS-stimulated HT-29 cell. Data were expressed as mean ± SEM (*n* = 4, * p < 0.05).

Akt1 among other subtypes of Akt (Akt2 and Akt3) is involved in various cancers [28]. For the reason we searched potential miRNA for regulating Akt using some bioinformatics algorithms. DIANA Lab and miRanda algorithms identified one of the most relevant miRNAs: miR-149. MiR-149 functions as a tumor suppressor *in vivo* [29, 30]. Akt1 3′-UTR is complementary to the “seed sequence” of miR-149 (Figure 4A). To verify this predictions, Akt1 3′-UTR was cloned and transfected in psiCheck-2 dual-luciferase reporter vector. The results implied that miR-149 repressed luciferase activity with a luciferase reporter plasmid containing sites of the Akt 3′-UTR (Figure 4B). Furthermore, we found that BPIS reduced Akt phosphorylation and subsequently activated the NF-κB-p65, while pretreatment with the miR-149 inhibitor attenuated BPIS-inhibited total Akt, Akt phosphorylation and NF-κB-p65 expression (Figure 4C). Thus, we further investigated that miR-149 inhibitor significantly reversed the inhibited IL-1β and IL-6 expression by BPIS, through the mediation of Akt dephosphorylation in LPS-stimulated HT-29 cells. Simultaneously, BPIS-upregulated IL-10 was reversed by miR-149 inhibitor (Figure 4D). Overall, this data provides experimental evidences that Akt is a direct target gene of miR-149.

### BPIS reduces upregulated miR-149 by ROS accumulation and exhibits anti-inflammatory activities

**Figure 5:**
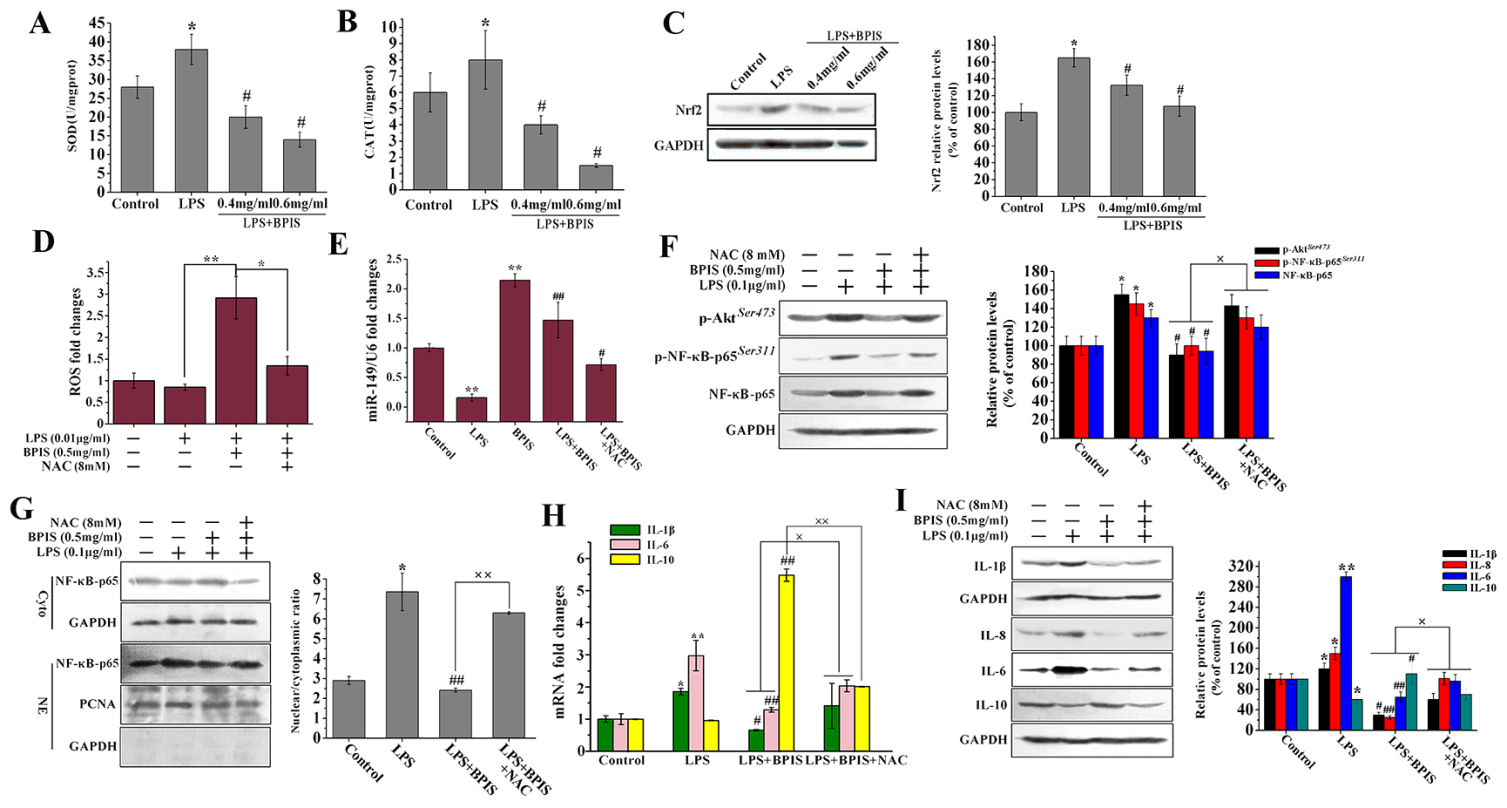
BPIS accelerated ROS production against inflammatory response in HT-29 cells **(A-C)** Cells untreated and treated with 0.01ug/ml LPS alone or treated by adding 0.01 ug/ml LPS with 0.4 mg/ml BPIS and 0.6 mg/ml BPIS. The SOD and CAT activities were measured by enzyme activity testing. The protein expression levels of Nrf2 were measured by western blot. Columns were expressed as mean ± SEM (*n* = 3, *p<0.05 vs. control, # p<0.05 vs. LPS). **(D)** Cells untreated and treated with 0.01ug/ml LPS alone or treated by adding 0.01 ug/ml LPS with 0.5 mg/ml BPIS and 8 mM NAC (ROS inhibitor). The ROS generation was measured by microplate reader assay. Each bar represents the mean ± S.D. of three independent experiments. *p<0.05, **p<0.01. **(E)** qRT-PCR analysis of miRNA -149 expression in treated 0.01ug/ml LPS or 0.5 mg/ml BPIS alone cells and treated by adding 0.01ug/ml LPS with 0.5 mg/ml BPIS cells or pre-incubated with 8 mM NAC 30 min before adding LPS with BPIS cells for 24 h. Columns were expressed as mean ± SEM (*n* = 3, **p<0.01 vs. control, # p<0.05 vs. LPS, ## p<0.01 vs. LPS). **(F)** Cells were pre-incubated with 8 mM NAC 30 min before adding 0.01ug/ml LPS with 0.5 mg/ml BPIS for 24 min. Western blot analysis was performed to determine Akt phosphorylation, NF-κB-p65 phosphorylation and NF-κB-p65. Columns were expressed as mean ± SEM (*n* = 3, *p<0.05 vs. control, # p<0.05 vs. LPS, ×p<0.05). **(G)** Cells were pre-incubated with 8 mM NAC 30 min before adding 0.01ug/ml LPS with 0.5 mg/ml BPIS for 24 h. Western blot assays of NF-κB-p65 expression in nuclear and cytosolic. PCNA and GAPDH were the loading controls, and the absence of GAPDH in nuclear extracts indicated no cytoplasmic protein. Columns were expressed as mean ± SEM (*n* = 3, *p<0.05 vs. control, ## p<0.01 vs. LPS, ××p<0.01). **(H)** qRT-PCR analysis of IL-1β, IL-6 and IL-10 mRNA in treated LPS alone cells and treated by adding LPS with 0.5 mg/ml BPIS cells or pre-incubated with 8 mM NAC 30 min before adding 0.01ug/ml LPS with 0.5 mg/ml BPIS cells for 24 h. **(I)** Cells were incubated with 0.5 mg/ml BPIS in presence or absence of 8 mM NAC and stimulated with 0.01ug/ml LPS for 24 h. The protein expression levels of IL-1β, IL-6, IL-8 and IL-10 were measured by western blot. Columns were expressed as mean ± SEM (*n* = 3, *p<0.05 vs. control, **p<0.01 vs. control, # p<0.05 vs. LPS, ## p<0.01 vs. LPS, ×p<0.05).

Increased oxidative damages, if not repaired, can induce chronic inflammation. Consequently, leads to the progression of inflammatory diseases [31-33]. BPIS could apparently suppress the expression of Nrf2, then reduce SOD and CAT activities, and ultimately result in the ROS accumulation (Figure 5A-5C). To investigate the possible involvement of ROS in BPIS-induced anti-inflammatory activities, ROS production followed by BPIS treatment was tested in LPS-induced HT-29 cells. The results showed that BPIS significantly (*p*<0.01) enhanced the intracellular ROS accumulation and miR-149 expression (Figure 5D and 5E). As expected, NAC, an inhibitor of ROS, noticeably reversed miR-149 upregulation and ROS cumulation of BPIS-induced (Figure 5D and 5E), including BPIS-inhibited the expression of IL-1β, IL-6 and IL-10 in both RNA and protein level (Figure 5H and 5I). To provide further insight into the anti-inflammatory mechanism of ROS, we testified that NAC has reversed the blockade of BPIS-induced Akt/NF-κB signaling pathway (Figure 5F and 5G). Hence, the inhibitory action of BPIS on miR-149 expression in response to LPS stimulation is ROS dependent.

### Suppressive effects of BPIS on inflammatory activities in xenograft mice models

**Figure 6:**
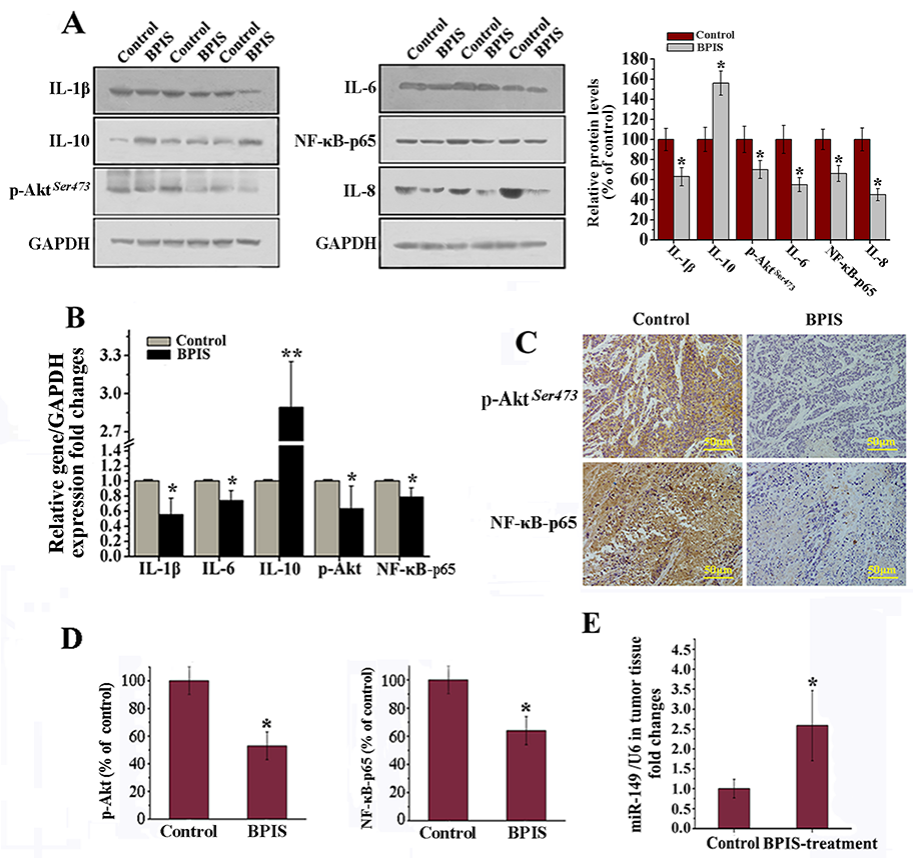
BPIS attenuated inflammatory responses in colon cell xenograft tumor models Colon cancer cells were implanted subcutaneously in the right flank of male nude mice. **(A)** Western blot analysis. Effects of BPIS on expression of p-Akt, NF-κB-p65 and inflammatory cytokines protein levels. Columns were expressed as mean±SEM (*n* = 3, *p<0.05 vs. control). **(B)** Relative gene expression levels were normalized to GAPDH expression levels by qRT-PCR. **(C)** Immunohistochemical analysis of the expression of Akt phosphorylation and NF-κB-p65 in colon cancer tissues by BPIS treatment group or control group. **(D)** Columns were expressed as mean±SEM (*n* = 3, *p<0.05 vs. control). **(E)** qRT-PCR analysis of miR-149 *in vivo*. Data were presented as mean ± SEM (n=10, *p<0.05 vs. control).

The anti-inflammatory activities of BPIS were tested in bare mice, which were implanted xenografts of HT-29 cells and received intraperitoneal injections of 1.0 mg BPIS/g for every two days. Based on the previous findings, the results indicated that proinflammatory factors levels were significantly reduced, and the antiinflammatory factor level was found increased as compared to the control groups (Figure 6A-6B). Of note, the expression levels of p-Akt and NF-κB-p65 were detected significantly (p<0.05) reduced in BPIS-treated tumor tissues (Figure 6C and 6D). However, more importantly we noticed considerable increased in the miR-149 expression level in BPIS-treated bare mice (Figure 6E). The results are consistent with the data from *in vitro* experiments and indirectly reveal their anti-inflammatory activities without any adverse effects *in vivo*.

## DISCUSSION

Enduring inflammatory responses resulted from overproduction of inflammatory mediators, leading to various inflammatory diseases including cancer [34]. Initiating and sustaining various inflammatory responses are regulated by pro-inflammatory molecules (IL-1β, IL-6 and IL-8) and anti-inflammatory cytokines (IL-10) [35]. In the present study, the capability and mechanism of BPIS to reduce inflammatory responses was investigated. We have found that IL-1β and IL-6 were downregulated, particularly IL-6 production was more strongly inhibited and IL-10 was upregulated by the BPIS treatment of LPS-induced HT-29 cells. However, TNF-α was unchanged. Therefore, we hypothesized that BPIS significantly reduce the level of IL-6 via suppressing the levels of NF-κB and STAT3 in the same time. Because it is well known that IL-6 was significantly elevated due to STAT3 and NF-κB activation [36]. However, TNF-α has no markable changes by BPIS treatment. Some studies show that mutant p53 amplifies the aggressive behavior of cancer cells exposed to inflammatory cytokines and TNF-α. Furthermore, HT-29 is a p53 mutant colon cancer cell line. We speculated that TNF-α has no markable changes by BPIS treatment, because mutant P53 enhanced TNF-a secretion [37]. Importantly, *in vivo* experiments showed that BPIS displayed the same effects in a xenograft mice model (Figure 6).

Cancer cells usually have a higher level of ROS, and over-expression of antioxidant enzymes in comparison with normal cells. Therefore, cancer cells are more potent to break ROS threshold by producing additional ROS, which lead to the apoptosis of cancer cell. Our previous study also showed that we could obtain 50mg of BPIS from 2g of foxtail millet bran and BPIS can induce the accumulation of ROS [9]. ROS have been independently linked to both inflammation and miRNA regulation [38, 39]. More recently, miRNAs caught a considerable attention of scientists, partly because of their key contribution of in understanding of anti-inflammatory effects of multiple drugs [40]. Nevertheless, the role of ROS, miRNA and various inflammation factors as well as their relationship in the development of CRC remains poorly understood. Previous studies reported a downregulation of miR-149 in NSCLC cells, and the expression of miR-149 was inversely correlated with the EMT phenotype of NSCLC cells. Hence, miR-149 functioned as a tumor suppressor [41]. Moreover, Our previous study found that BPIS could restrain the growth of tumor tissue. In addition, the expression of miR-149 was significantly increased in BPIS-treated tumor tissues. Therefore, we speculate that miR-149 may play a positive role in tumor suppression resulted from BPIS treatment *in vitro* and *in vivo* (Figure 4 and 6). Furthermore, BPIS induced miR-149 up-regulation in HT-29 cells was attenuated by inclusion of ROS inhibitor NAC in the treatment step (Figure 5D) suggesting that ROS played an important role in BPIS induced miR-149 up-regulation. Subsequently, we identified that Akt is a target gene of miR-149 (Figure 4). These results prove that BPIS activates the existence of an ROS/miRNA-149/anti-inflammation framework.

Inflammation promotes tumorigenesis by activating NF-κB and MAPK pathways [42, 43]. The atypical increased in Akt phosphorylation might contribute to the development of inflammation [44]. In this study, BPIS strongly inhibits the activation of NF-κB and NF-κB-mediated inflammatory responses by suppressing the nuclear translocation of NF-κB and the total expression of NF-κB in LPS-stimulated HT-29 cells (Figure 2). Furthermore, our study also showed that BPIS inhibited NF-κB expression by suppressing Akt phosphorylation in LPS-induced HT-29 cells, and pretreatment with Akt phosphorylation activator IGF-1 abolished BPIS’s inhibitory effect (Figure 3). These results implicate that Akt, a target gene of miR-149, is necessary for NF-κB-mediated inflammation.

**Figure 7:**
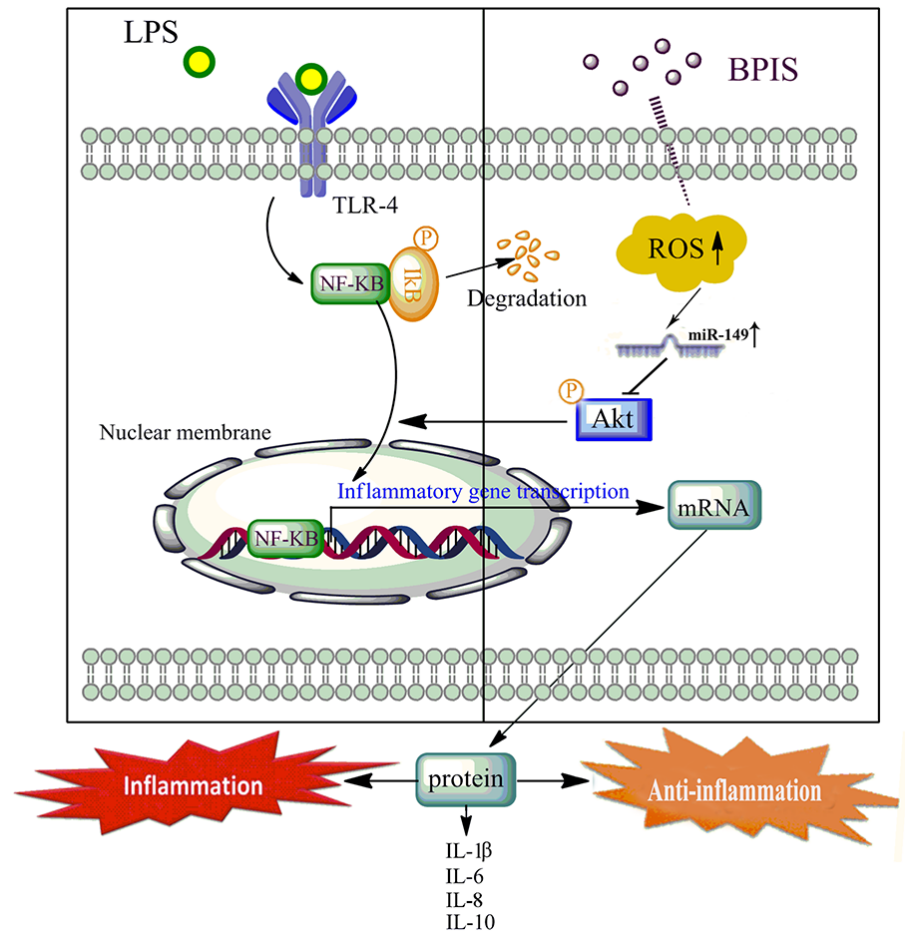
Proposed mechanism of the anti-inflammatory effects by BPIS induced LPS directly promoted the TLR4 activation, triggering its inflammatory pathways. BPIS treatment promoted the ROS accumulation, and led to the increase of miR-149 expression. Further, miR-149 directly targeted the 3′-UTR of Akt to block NF-kB nuclear translocation, and then attenuated expression of pro-inflammatory factors (IL-1β, IL-8 and IL-6) in LPS-induced HT-29 cells.

In conclusion, BPIS inhibits the initiation and progression of CRC by ameliorating the inflammatory microenvironment, and the presence of a signaling cascades of ROS / miR-149 / Akt / NF-κB axis for BPIS-mediated resistance to inflammation in LPS-induced HT-29 cells (Figure 7). Our results highlight BPIS as a potential pro-oxidant agent against inflammation and provide a future perspective to elucidate other molecular mechanisms involving BPIS and additional miRNAs in CRC and other types of cancer.

## MATERIALS AND METHODS

### Reagents and antibodies

DMEM/F-12 (v/v = 1:1) medium and fetal bovine serum (FBS) were purchased from GIBCO (Grand Island, NY, USA). LPS derived from Escherichia coli strain 055:B5; 4′,6-diamidino-2-phenylindole (DAPI); N-acetylcysteine (NAC) and insulin-like growth factor-1 (IGF-1, PI3K/Akt activator) were from Sigma (St. Louis, MO, USA). Enzyme-linked immunosorbent assay (ELISA) kits for tumor necrosis factor (TNF-α), interleukin-1β (IL-1β), interleukin-6 (IL-6), interleukin-8 (IL-8) and interleukin-10 (IL-10) were obtained from Shanghai Westang Bio-Tech (Shanghai, China). RNAiso Plus, PrimeScript RT MasterMix and SYBR Green PCR Master Mix were obtained from Takara (Shiga, Japan). 5- (and-6)-carboxy-2′, 7′-dichlorodihydrofluorescein diacetate (DCFDA) were obtained from Molecular Probe (Eugene, OR, USA). The nuclear and cytoplasmic protein extraction kit was from Beyotime Biotech (Beijing, China). The BCA protein kit was purchased from Thermo Fisher (Shanghai, China). Antibodies for TNF-α, IL-1β, and NF-κB were from Bioworld Technology (Minneapolis, MN, USA); Antibodies against total Akt, p-Akt (Ser 473), IL-6, IL-8, p-NF-κB-p65(Ser311) and IL-10 were purchased from Cell Signaling Technology Company (Shanghai, China). Antibodies for GAPDH and PCNA were from Abmart (Arlington, MA, USA).

### Extraction of bound polyphenols compounds from foxtail millet bran

Seeds of foxtail millet were purchased from a local company (Tian-xia-gu Limited Company, China). These seeds were cleaned with double distilled water and pulverized in a plate mill (THU 35B, Satake, Japan) to produce whole flour. The whole flour contains inedible bran and was sieved through a 44 μm mesh sieve to obtain a uniform fraction of bran [11]. The bran fraction was stored in airtight containers. Bound polyphenols of foxtail millet bran samples were extracted with some modifications of [45]. Briefly, 2 g foxtail millet bran flour were mixed with 50 ml 80 % (v/v) acetone for 10 min, followed by centrifugation at 1157 g for 5 min at 4 °C. The supernatant was discarded and the residue was repeated for two times. The residue of obtained after extraction of acetone was hydrolyzed with 2 M NaOH at room temperature for 1h. The mixture was neutralized with an appropriate amount of concentrated hydrochloric acid. To removing lipids, the mixture was extracted for five times with hexane, followed by centrifugation for 10 min at 1575 g, and the supernatant was collected. Subsequently, the supernatant was extracted with ethyl acetate (1:1, v/v) for 10 min at 4 °C, and was centrifugated (1575 g, 5 min, 4 °C). Combined supernatants were evaporated to dryness under vacuum at 45 °C and freeze-dried under vacuum. The powder stored at -80°C until used.

### Cell culture and treatment

Human colon carcinoma cell line HT-29 was obtained from the Chinese Type Culture Collection and cultured in DMEM/F-12 (v/v = 1:1) medium supplemented with 10% (v/v) heat-inactivated fetal bovine serum (FBS) at 37°C in 5% CO_2_ incubator. All media were supplemented with 100 U/ml penicillin (Sigma; St. Louis, MO, USA) and 100 mg/ml streptomycin (Sigma). LPS and NAC were dissolved in phosphate buffer (0.01 M, pH 7.2).

### Nuclear protein extraction and western blot analysis

The nuclear and cytosolic proteins were extracted with a nuclear protein extraction kit as per manufacturer’s instruction.(Beyotime Biotechnology, China). HT-29 cells were plated at 1×10^7^ in 75 cm^2^ plates and then incubated with LPS (0.01ug/mL) in the presence or absence of BPIS for 24 h. Protein concentrations of nuclear, cytosolic and whole cell lysates were determined using the BCA protein assay kit, and 60 μg of cell lysates were subjected to 10% sodium dodecyl sulfate polyacrylamide gel electrophoresis (SDS–PAGE), transferred to polyvinylidene fluoride (PVDF) membranes (Immobilon, Millipore, Bedford, MA, USA). Membranes were firstly blocked for 1 h at room temperature with blocking solution (5 % fresh milk in TBS plus Tween-20) and incubated with a specific primary antibody overnight at 4°C. Finally, the PVDF membranes were washed and incubated with corresponding secondary antibodies for 2 h at room temperature. Blots were developed on membranes using enhanced chemoluminescence.

### Quantitative real-time PCR

HT-29 cells were treated with LPS (0.01 μg/ml) with or without various concentrations of BPIS for 24 h. Total RNA was extracted using TRIzol (Invitrogen, CA). 500 ng of total RNA from each sample was reversely transcribed to cDNA using Prime Script RT Master Mix. Quantitative real-time PCR was performed for 1 cycle at 95 °C for 30 s, and 40 cycles at 95 °C for 5 s, 64 °C for 34 s and a melt curve step using a Step One Plus Real-Time PCR System (Applied Biosystems). The cDNA was amplified using the following selective primers (5’ > 3’): human TNF-α, CGA GTG ACA AGC CTG TAG CC(forward) and TGA AGA GGA CCT GGG AGT AGAT-(reverse); human IL-1β, TTA CAG TGG CAA TGA GGA TG-(forward) and TGT AGT GGT GGT CGG AGA TT(reverse); human IL-6, CCT TCG GTC CAG TTG CCT TCT-(forward) and -CAG TGC CTC TTT GCT GCT TTC-(reverse); human IL-10, AGA ACC AAG ACC CAG ACA TCA(forward) and GCA TTC TTC ACC TGC TCC AC (reverse); human glyceraldehyde-3-phosphate dehydrogenase (GAPDH), TGT TCC AGT ATG ACT CCA CTC-(forward) and TCC ACC ACC CTG TTG CTG TA(reverse); human NF-κB, ATC TGC CG A GTG AAC CGA AACT-(forward) and CCA GCC TGG TCC CGT GAA A(reverse); human Akt1,ACT TCC CCC AGT TCT CCT ACT CG-(forward) and CCC ACA GCA CAA AAA CGT CTT TC-(reverse);

### Enzyme-linked immunosorbent assay (ELISA)

The study cells were plated at 2×10^5^ cells/well and then incubated with LPS (0.01 ug/mL) in the presence or absence of BPIS (0.6 mg/ml) for 24 h. The cell supernatants were collected and centrifuged at 13,000×g at 4°C for 5 min. TNF-α, IL-1β, IL-6, IL-10 in the supernatants were measured by ELISA as per manufacturer’s instruction (Westang Bio-Tech, China) and measured using Bio-Tek MQX200 (Bio-Tek Instruments Inc., Winooski, VT, USA). Final values were normalized to total protein concentrations.

### Detection of intracellular ROS generation

Detection of ROS was performed using the stain of DCFH-DA (Beyotime Institute of Biotechnology, Jiangsu, China) according to the manufacturer’s recommended protocol [39]. The nonfluorescent derivatives 2’, 7’- dichlorofluorescin diacetate (DCFHDA) has no fluorescence and can freely pass through cell membrane. After entering the cell, it can be hydrolyzed into DCFH by intracellular esterase. BPIS could promote the production of ROS, which oxidizes nonfluorescent DCFH into a fluorescent dichlorofluorescein (DCF). Detection of DCF fluorescence indicate the level of ROS in the cells. HT-29 cells were plated at 1×10^6^ cells/well and then incubated with LPS (0.01ug/mL) in the presence or co-treatment with BPIS for 24 h. After 24h, the old medium was removed, and cells were washed twice with phosphate buffered saline (PBS). Then the medium was replaced with serum-free DMEM/F-12 medium containing 1 ml of 1 μM DCFH-DA for 30 min at 37°C. Then the cells were harvested and washed three times with PBS. The fluorescence of the cell was read at excitation/emission wavelengths of 485/525nm in a fluorescence microplate reader (Thermo Fisher Scientific, Franklin, MA, USA).

### Constructs and plasmids

The 3′-UTR of Akt containing miR-149-binding sites was amplified and cloned into a psiCHECK2 vector to generate psiCHECK2-Akt. The psiCHECK2 vector contains two reporter gene (firefly and renilla luciferase) and is designed for end point lytic assays. Luciferase activity was measured 48 h later using the DualLuciferase reporter assay (Promega). Values were normalized with firefly luciferase activity.

### Transient transfection

Transient transfections were performed with Lipofectamine 2000 reagent (Invitrogen). MiRNA oligonucleotide transfections were performed as per established protocol. HT-29 cells were seeded into 6-well plates. Next, miR-149 inhibitor, or a matched miRNA negative control (miR-N.C) (Genepharma, Shanghai, China) was added to the culture media. After 6 h of transfection, the medium was replaced with DMEM/F-12, and cells were incubated for 24 h. Transfection efficiency was measured by confocal laser scanning microscope (Leica, TCS SP2, Germany).

### Luciferase reporter assays

Luciferase assays were performed according to the manufacturer’s protocol. HT-29 cells seeded into 6-well plates at a density of 8×10^4^ per well, were co-transfected with 50 ng psiCheck-2 with insertion of the wild-type 3′-UTR of Akt1 or empty vector, along with miR-149 inhibitors or negative control (final concentration, 50 nM). After 24 h transfection, cells incubated with LPS (0.01ug/mL) in the treatment of BPIS for 24 h and then were lysed and assayed for luciferase activities using the TransDetect Double-Luciferase Reporter Assay Kit (Transgen, Beijing, China). The data recorded on fluorescence microplate reader (Thermo Fisher Scientific, Franklin, MA, USA) were normalized by dividing Firefly luciferase activity with that of Renilla luciferase.

### Immunofluorescence staining

To show that BPIS inhibits the translocation of NF-κB, HT-29 cells (1 × 10^4^ cells/well) were cultured on cover slips and treated for 24 h by adding 0.01 μg/ml LPS with either 0.6 mg/ml BPIS. After treatment, cells were fixed with methanol for 15 min at 4 °C and then permeabilized with 0.1% Triton-X100 for 20 min. Next, the cells were blocked with 3% bovine serum albumin in PBS for 1 h. The samples were incubated with NF-κB antibody at 4°C overnight, followed by incubating with a FITC-conjugated goat anti-rabbit or antimouse antibody (1:300; Jackson ImmunoResearch, West Grove, PA) for 2 h at room temperature. The nucleus was stained with 0.1 μg/mL of 4′,6-diamidino-2-phenylindole (DAPI) for 10 min in the dark. The stained cells were examined by confocal laser scanning microscope (Leica, TCS SP2, Germany).

### Animals and immunohistochemical analysis

Achievement of animal tumour was carried out following procedures approved by the Institutional Animal Care and Use Committee of China Institute for radiation protection. The review board and ethics committee of the institution specifically approved this study. Twenty BALB/c male mice (five weeks old) were obtained from the Institute of Zoology, Chinese Academy of Sciences. HT-29 cells were injected subcutaneously into the right flank of each nude mouse and a solid tumour was apparent by 15 days. The mice were divided into two groups. Mice in the BPIS group received an intraperitoneal injection of 1.0 mg BPIS/g body weight every two days (total 7 injections), and the control mice were treated with physiological saline instead. After seven BPIS injections, all mice were sacrificed, and tumours were excised forimmunohistochemical analysis.

Immunohistochemistry was implemented as previously described [46]. The paraffin sections were de-waxed in xylene and dehydrated in concentration gradient ethanol. Antigen retrieval was carried out using citrate buffer (pH 6.0). All sections were stained with anti-human p-Akt and NF-κB. Staining was performed using a universal labeled streptavidin-biotin kit according to the standard protocol. The percentage of cells was assessed by counting the number of brown-stained cells in five random fields in each section.

### Data processing and statistical analysis

All data were presented as mean ± standard deviation (S.D.) for three independent experiments. Data were analyzed by using the one-way analysis of variance followed by Tukey’s multiple-range test with the SPSS 16.0 system. Differences were considered as statistically significant at p< 0.05.

## Abbreviations

FBS: fetal bovine serum
BPIS: bound polyphenols of inner shell
LPS: lipopolysaccharide
ROS: reactive oxygen species
TNF-α: tumor necrosis factor α
IL-1β: interleukin-1β
IL-6: interleukin-6
IL-8: interleukin-8
IL-10: interleukin-10
NAC: N-acetylcysteine
ELISA: enzyme-linked immunosorbent assay
SDS-PAGE: Sodium dodecyl sulfate polyacrylamide gel electrophoresis
PVDF: polyvinylidene fluoride
IGF-1: insulin-like growth factor-1
DAPI: 4′,6-diamidino-2-phenylindole
DCFDA: 5- (and-6)-carboxy-2′, 7′-dichlorodihydrofluorescein diacetate
NF-κB: nuclear factor-kappaB
